# Cluster-efficient pangenome graph construction with nf-core/pangenome

**DOI:** 10.1093/bioinformatics/btae609

**Published:** 2024-10-14

**Authors:** Simon Heumos, Michael L Heuer, Friederike Hanssen, Lukas Heumos, Andrea Guarracino, Peter Heringer, Philipp Ehmele, Pjotr Prins, Erik Garrison, Sven Nahnsen

**Affiliations:** Quantitative Biology Center (QBiC) Tübingen, University of Tübingen, Tübingen, 72076, Germany; Biomedical Data Science, Department of Computer Science, University of Tübingen, Tübingen, 72076, Germany; M3 Research Center, University Hospital Tübingen, Tübingen, 72076, Germany; Institute for Bioinformatics and Medical Informatics (IBMI), Eberhard-Karls University of Tübingen, Tübingen, 72076, Germany; University of California, Berkeley, Berkeley, CA 94720, United States; Quantitative Biology Center (QBiC) Tübingen, University of Tübingen, Tübingen, 72076, Germany; Biomedical Data Science, Department of Computer Science, University of Tübingen, Tübingen, 72076, Germany; M3 Research Center, University Hospital Tübingen, Tübingen, 72076, Germany; Institute for Bioinformatics and Medical Informatics (IBMI), Eberhard-Karls University of Tübingen, Tübingen, 72076, Germany; Department of Computational Health, Institute of Computational Biology, Helmholtz Munich, Munich, 85764, Germany; Comprehensive Pneumology Center with the CPC-M bioArchive, Helmholtz Zentrum Munich, Member of the German Center for Lung Research (DZL), Munich, 81377, Germany; TUM School of Life Sciences Weihenstephan, Technical University of Munich, Freising, 81377, Germany; Department of Genetics, Genomics and Informatics, University of Tennessee Health Science Center, Memphis, TN 38163, United States; Human Technopole, Milan 20157, Italy; Quantitative Biology Center (QBiC) Tübingen, University of Tübingen, Tübingen, 72076, Germany; Biomedical Data Science, Department of Computer Science, University of Tübingen, Tübingen, 72076, Germany; M3 Research Center, University Hospital Tübingen, Tübingen, 72076, Germany; Institute for Bioinformatics and Medical Informatics (IBMI), Eberhard-Karls University of Tübingen, Tübingen, 72076, Germany; Department of Computational Health, Institute of Computational Biology, Helmholtz Munich, Munich, 85764, Germany; Department of Genetics, Genomics and Informatics, University of Tennessee Health Science Center, Memphis, TN 38163, United States; Department of Genetics, Genomics and Informatics, University of Tennessee Health Science Center, Memphis, TN 38163, United States; Quantitative Biology Center (QBiC) Tübingen, University of Tübingen, Tübingen, 72076, Germany; Biomedical Data Science, Department of Computer Science, University of Tübingen, Tübingen, 72076, Germany; M3 Research Center, University Hospital Tübingen, Tübingen, 72076, Germany; Institute for Bioinformatics and Medical Informatics (IBMI), Eberhard-Karls University of Tübingen, Tübingen, 72076, Germany

## Abstract

**Motivation:**

Pangenome graphs offer a comprehensive way of capturing genomic variability across multiple genomes. However, current construction methods often introduce biases, excluding complex sequences or relying on references. The PanGenome Graph Builder (PGGB) addresses these issues. To date, though, there is no state-of-the-art pipeline allowing for easy deployment, efficient and dynamic use of available resources, and scalable usage at the same time.

**Results:**

To overcome these limitations, we present *nf-core*/*pangenome*, a reference-unbiased approach implemented in Nextflow following nf-core’s best practices. Leveraging biocontainers ensures portability and seamless deployment in High-Performance Computing (HPC) environments. Unlike PGGB, nf-core/pangenome distributes alignments across cluster nodes, enabling scalability. Demonstrating its efficiency, we constructed pangenome graphs for 1000 human chromosome 19 haplotypes and 2146 *Escherichia coli* sequences, achieving a two to threefold speedup compared to PGGB without increasing greenhouse gas emissions.

**Availability and implementation:**

nf-core/pangenome is released under the MIT open-source license, available on GitHub and Zenodo, with documentation accessible at https://nf-co.re/pangenome/docs/usage.

## 1 Introduction

The availability of high-quality population-wide whole-genome assemblies (Liu *et al.* 2020, Leonard *et al.* 2022, Zhou *et al.* 2022, Kang *et al.* 2023, Liao *et al.* 2023, Weller *et al.* 2023) offers new opportunities to study sequence evolution and variation within and between genomic populations. A challenge is simultaneously representing and analyzing hundreds to thousands of genomes at a gigabase scale. One solution here is a pangenome. It models a population’s entire set of genomic sequences (Ballouz *et al.* 2019). In contrast to reference-based genomic approaches, which relate sequences to a linear genome, pangenomics relates each new sequence to all the others represented in the pangenome (The Computational Pan-Genomics Consortium 2018, Eizenga *et al.* 2020, Sherman and Salzberg 2020) minimizing reference-bias. Pangenomes can be described as sequence graphs which store DNA sequences in nodes with edges connecting the nodes as they occur in the individual sequences (Hein 1989). Genomes are encoded as paths traversing the nodes (Garrison *et al.* 2018).

Current pangenome graph construction methods exclude complex sequences or are reference-biased (Minkin *et al.* 2017, Chin *et al.* 2023). One recent approach that overcomes such limitations is the PanGenome Graph Builder (PGGB) pipeline (Garrison *et al.* 2024). PGGB iteratively refines an all-to-all whole-genome alignment graph that lets us explore sequence conservation and variation, infer phylogeny, and identify recombination events. PGGB has been extensively evaluated (Andreace *et al.* 2023, Garrison *et al.* 2024) and applied to build the first draft human pangenome reference (Liao *et al.* 2023). However, PGGB is implemented in bash, which (a) makes it difficult to deploy on High-Performance Computing (HPC) systems, (b) does not allow for a fine granular tuning of computing resources for different steps of the pipeline (Sztuka *et al.* 2024), and (c) limits its cluster scalability to one node. These limitations greatly hinder the broad application of large-scale pangenomes.

To compensate for that, we wrote *nf-core*/*pangenome*, a reference-unbiased approach to construct pangenome graphs. Mirroring PGGB, nf-core/pangenome is implemented in Nextflow (Di Tommaso *et al.* 2017). In contrast to PGGB, nf-core/pangenome can distribute the quadratic all-to-all base-level alignments across nodes of a cluster by splitting the approximate alignments into problems of equal size. We benchmarked the time spent on base-pair level alignments and show that it is reduced linearly with an increase in alignment problem chunks (Supplementary Material 5.5). We showcase the workflow’s scalability by applying it to 1000 chromosome 19 human haplotypes and 2146 *Escherichia coli* sequences, which were built in less than half the time PGGB required while not increasing the CO2 equivalent (CO2e) emissions (Lannelongue *et al.* 2021).

## 2 Materials and methods

### 2.1 Pipeline overview

**Figure 1.**
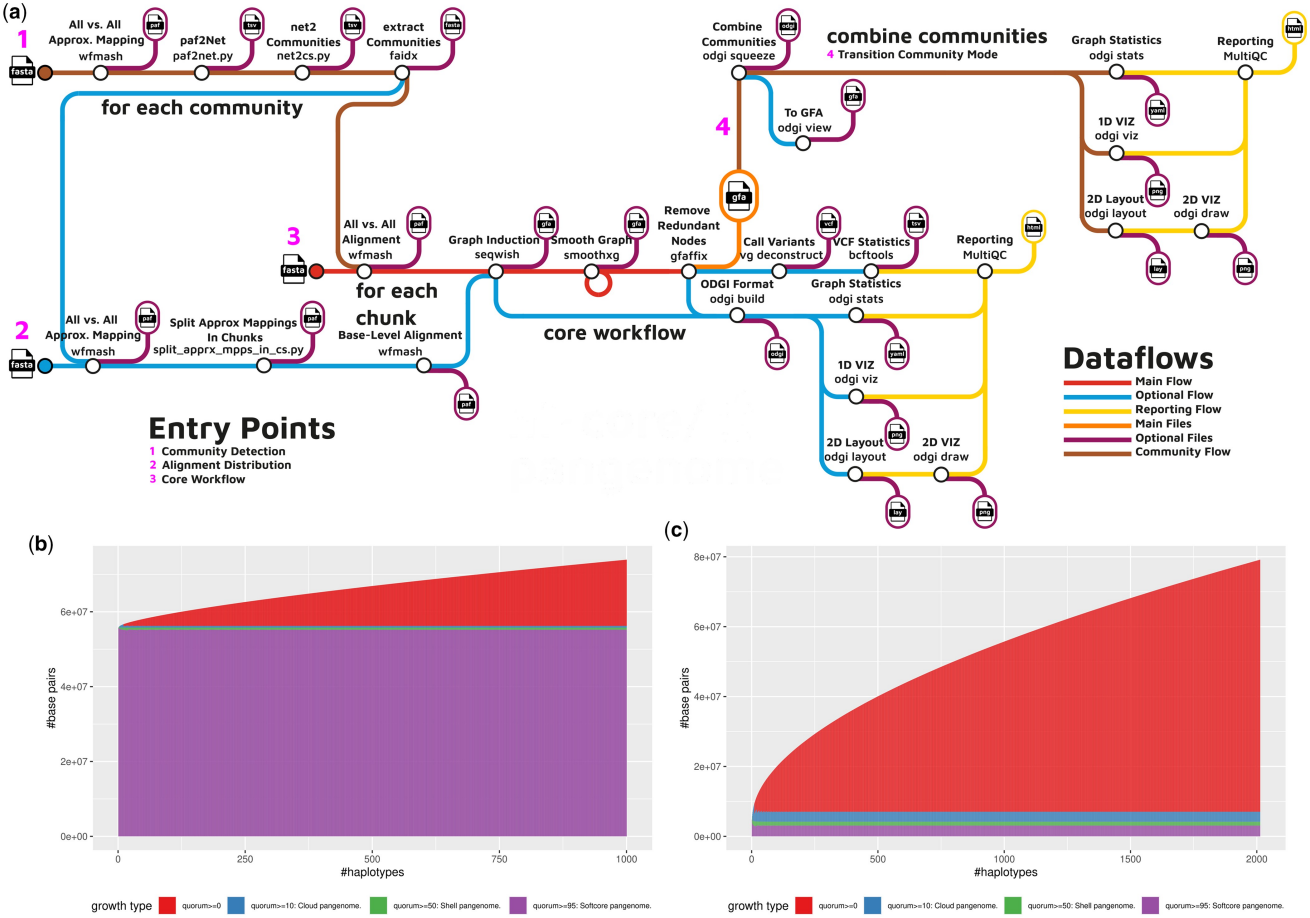
(a) Schematic representation of the nf-core/pangenome workflow processes and detailed analysis steps. The input consists of one FASTA file containing all sequences. The pipeline comes with three major entry points: (1) community detection, which identifies clusters of related sequences or regions in the pangenome graph to reveal biologically significant patterns like conserved or divergent areas across genomes (Supplementary Material 5.2), (2) alignment distribution, and (3) core workflow. Optional community detection (1) is performed on the input sequences. If selected, the heavy all-to-all base-pair level alignments (2) can be split into problems of equal size. nf-core/pangenome’s core workflow (3) is a direct mirror of PGGB. If running in community mode, all communal graphs are combined into one (4) and the subsequent quality control subworkflow is executed. The output is a pangenome graph in GFA format. (b, c) Pangenome growth curves of the built pangenome graphs. Growth type is defined as the minimum fraction of haplotypes that must share a graph feature after each time a haplotype is added to the growth histograph. quorum >=0: all sequences without any filtering are considered. quorum >=10: sequences traversed by at least 10% of the haplotypes. quorum >=50: sequences traversed by at least 50% of haplotypes. quorum >=95: sequences traversed by 95% of haplotypes. (b) Pangenome growth curve of the chromosome 19 pangenome graph of 1000 haplotypes. (c) Pangenome growth curve of the *Escherichia coli* pangenome graph of 2013 haplotypes.

The pipeline’s (Fig. 1a) input is a FASTA file compressed with *bgzip* (Li *et al.* 2009) containing the sequences to create the graph. Sequence names should follow the Pangenome Sequence Naming specification (PanSN-spec) (https://github.com/pangenome/PanSN-spec, last accessed October 2024). The primary output is a pangenome variation graph (Garrison *et al.* 2018) in the Graphical Fragment Assembly (GFA) format version 1 (http://gfa-spec.github.io/GFA-spec/GFA1.html, last accessed October 2024).

#### 2.1.1 Core workflow

The core workflow of nf-core/pangenome mirrors PGGB (Fig. 1a) with additional enhancements: (a) All concurrent processes can be run in parallel. (b) Each process can be given individual computing resources.

The pipeline begins with an all-to-all alignment of the input sequences using the whole-chromosome pairwise sequence aligner WFMASH (https://github.com/waveygang/wfmash, last accessed October 2024), avoiding reference, order, or orientation bias, allowing every sequence to serve as a reference. In the pangenome graph induction step SEQWISH (Garrison and Guarracino 2023), an alignment to variation graph inducer, converts the sequence alignments into a variation graph. The graph is then simplified using SMOOTHXG (Garrison *et al.* 2024): A 1-dimensional (1D) graph embedding (Heumos *et al.* 2024) orders the graph’s nodes to best match the nucleotide distances of the genomic paths of the graph. Next, the graph is split into partially overlapping segments. The sequences of each segment are realigned with a local Multiple Sequence Alignment (MSA) kernel, partial order alignment (POA) (Lee *et al.* 2002). Afterwards, the segments are laced back together into a variation graph. By default, the SMOOTHXG process is applied 3 times in order to smooth the edge effects at the boundaries of the segments. Finally, we employ GFAFFIX (Liao *et al.* 2023) to systematically condense redundant nodes within the graph.

Graph quality is assessed with ODGI (Guarracino *et al.* 2022), which provides statistics and visualizations. Optionally, variants can be called against any (reference) path(s) in the graph using *vg deconstruct* (Garrison *et al.* 2018). Results are summarized in a MultiQC (Ewels *et al.* 2016) report. Pipeline implementation details are given in Supplementary Material 5.1.

If desired, the pipeline performs community detection to identify clusters of related sequences in the pangenome graph, revealing biological patterns such as conserved or divergent regions across genomes (Supplementary Material 5.2), with the core workflow executed for each community in parallel.

## 3 Results

### 3.1 Building a 1000 haplotypes chr19 pangenome graph

The Human Pangenome Resource Consortium (HPRC) recently built a draft human pangenome of 90 haplotypes. However, haplotype data for thousands of individuals was already generated by the 1000 Genomes Project (1KGP) (The 1000 Genomes Project Consortium 2010). As a use case, we used nf-core/pangenome to build a graph of 1000 chromosome 19 haplotypes (Kuhnle *et al.* 2020) in 3 days, emitting 51.07 kg CO2e. PGGB took 7 days for the same task (56.32 kg CO2e). In Fig. 1b the pangenome growth curve generated with PANACUS (Liao *et al.* 2023) shows nucleotide growth as more haplotypes are added. The softcore pangenome, defined as sequences traversed by 95% of haplotypes, comprises the majority of the pangenome even with 1000 haplotypes. This stability may be due to the exclusion of complex regions like the centromere in the short-read data.

### 3.2 Building a 2146 sequences *E. coli* pangenome graph

To evaluate the pipeline’s scalability, we built a pangenome graph of 2146 *E. coli* sequences. The nf-core/pangenome graph was completed in 10 days, emitting 175.18 kg CO2e, while PGGB could not finish within 30 days due to cluster time restrictions. For the growth curve (Fig. 1c) we excluded 130 plasmid sequences. The softcore pangenome remains stable at ∼3Mb, but its size constitutes less than 10% of the total pangenome. This substantial pangenomic growth is likely driven by horizontal gene transfer, as bacteria incorporate genes from one another at various genomic locations. Other reasons could be sequencing errors or human contamination (Breitwieser *et al.* 2019).

## 4 Discussion

We implemented nf-core/pangenome, an easy-to-install, portable, and cluster-scalable pipeline for unbiased pangenome variation graph construction. It is the first pangenomic pipeline within the nf-core framework that enables the comparative analysis of gigabase-scale pangenome datasets. While tools like Minigraph (Li *et al.* 2020) or PGR-TK (Chin *et al.* 2023) also address pangenome analysis, nf-core/pangenome uniquely integrates this capability into the standardized nf-core framework, offering compatibility with a wide range of modular workflows and community-developed best practices.

The pipeline’s core workflow has been successfully applied to *Neisseria meningitidis* (Yang *et al.* 2023), wild grapes (Cochetel *et al.* 2023), humans (Guarracino *et al.* 2023, Liao *et al.* 2023), grapevines (Guo *et al.* 2024), taurines (Milia *et al.* 2024), and rats (Villani *et al.* 2024) underpinning the community effort to focus on a best-practice workflow to create reference-unbiased and sequence complete pangenome graphs. The modular domain-specific language (DSL) 2 pipeline structure facilitates easy exchange of processes with alternative tools, expanding its functionality and integration with other (sub-)workflows.

We have shown that we are able to perform all-vs-all base pair level alignments of thousands of sequences. When executed on an HPC, nf-core/pangenome’s parallel workflow accelerates graph construction compared to PGGB. PGGB’s inability to assign individual computational resources to each pipeline step leads to the allocation of one whole node of an HPC, despite the fact that some processes can only make use of one thread. This blocks valuable CPU cycles. In contrast, nf-core/pangenome leverages Nextflow’s process management for optimal resource allocation, crucial for cloud-based executions.

Competing pipelines either lack workflow management system (Chin *et al.* 2023), or their workflow language of choice is e.g. Toil (Vivian *et al.* 2017, Hickey *et al.* 2024) which makes them less user-friendly, less cluster-efficient, and less portable (Wratten *et al.* 2021). nf-core/pangenome is currently the only pangenomics pipeline that is optionally monitoring its CO2 footprint. The measurements have shown that constructing extensive pangenome graphs, such as the 2146 *E. coli* graph, requires a considerable amount of energy. Therefore, we recommend assessing the rationale and methodology before conducting energy-intensive experiments.

Although we expect our pipeline to scale for future challenges, such as for the next HPRC phase which targets 350 individuals, further optimizations are possible: The IMplicit Pangenome Graph (IMPG) (https://github.com/ekg/impg, last accessed October 2024) tool extracts homologous loci from genomes mapped to a specific target region. This would allow us to break the whole genome multiple alignments into smaller pieces, construct a pangenome graph for each piece, and lace these together into a full graph with gfalace (https://github.com/pangenome/gfalace, last accessed October 2024).

We anticipate the pipeline, or its parts, will enhance current single linear reference analysis methods to explore whole population variation instead of focusing on one reference only. Looking ahead, pangenome construction pipelines like nf-core/pangenome will play a pivotal role in studying entire populations, single-cell whole genome sequencing analysis, and constructing personalized (medical) pangenome references (Sirén *et al.* 2024).

## Supplementary Material

btae609_Supplementary_Data

## Data Availability

Code and data resources for this manuscript and its figures are available in the public repository: https://github.com/subwaystation/pangenome-paper.
