# Supplementary material for: Cluster-efficient pangenome graph construction with nf-core/pangenome
Source: Bioinformatics. 2024 Oct 14;40(11):btae609. doi: 10.1093/bioinformatics/btae609 (PMC11568064; doi:10.1093/bioinformatics/btae609)
Supplement: btae609_Supplementary_Data [file btae609_supplementary_data.pdf]

5 Supplement

5.1 Implementation

nf-core/pangenome is written in Nextflow using its latest domain-specific language (DSL) 2 syntax which facilitates a modular pipeline structure. Each software tool is an individual process that is implemented in its own module (https://nf-co.re/docs/contributing/modules, last accessed October 2024). Processes are concatenated into subworkflows (https://nf-co.re/docs/contributing/subworkflows, last accessed October 2024). Developed with the nf-core framework, the pipeline follows a set of best-practice guidelines ensuring high-quality development, maintenance, and testing standards. Specifically, we provide community support via a dedicated Slack channel (https://nfcore.slack.com/channels/pangenome, last accessed October 2024), GitHub issues, and detailed documentation (https://nf-co.re/pangenome/1.1.2/docs/usage, last accessed October 2024).

Versioning and portability are enabled through (a) semantic versioning (https://semver.org/, last accessed October 2024) of the pipeline via tagged releases on GitHub, (b) packaging software dependencies in archivable containers so that the software compute environment is the same across different systems, and (c) summarizing software versions and parameters in the MultiQC report of the pipeline. nf-core/ pangenome uses biocontainers to facilitate portability across different computing resources like HPC clusters, cloud platforms, or local machines. Code changes are evaluated with GitHub Actions' continuous integration (CI) using a pipeline-specific small test data set. For each new pipeline release, a full-size test is run on Amazon Web Services (AWS) validating the code integrity and cloud compatibility of real-world data sets. Specifically, a pangenome graph is created from the 8 Saccharomyces cerevisiae strains of the Yeast Population Reference Panel (YPRP) (Yue and Liti 2018). The results of such a run are available on the nf-core webpage (https://nf-co.re/pangenome/1.1.2/results/pangenome/results-0e8a38734ea3c0397f94416a0146a2972fe2db8b, last accessed October 2024). Because we implemented our processes using DSL2 nf-core/modules (https://github.com/nf-core/modules, last accessed October 2024), they can be distributed easily to other users to share commonly used processes or subworkflows across pipelines. This boosts the reuse of existing work done by the community to be integrated into future pipelines.

5.2 Chromosome community detection

Chromosome community detection involves identifying clusters of closely related sequences or genomic regions within the pangenome graph. These communities represent areas of high similarity or shared sequence variants across multiple genomes. Detecting such communities allows researchers to focus on biologically meaningful patterns, such as conserved or highly divergent regions, which are crucial for understanding genetic diversity, evolutionary relationships, and genotype-phenotype associations.

For example, eukaryotic genomes are typically organized into chromosomes, and this organization is considered during graph construction in community detection mode. Chromosome groupings from the input sequence are examined, and homologies detected in the all-to-all WFMASH mapping step are passed through the Leiden clustering algorithm (Traag *et al.*, 2019), where the edge weight is calculated as $mapped\_length * mapped\_identity$. The nf-core/pangenome core workflow is then executed in parallel for each of the resulting communities. The communal graphs are subsequently merged into a single graph, followed by a final round of quality control (see Fig 1a, brown tubes).

This approach works effectively for large input sequences with a mapping length filter greater than 1 Mb, as demonstrated by Guarracino *et al.* (2023) when exploring recombination between heterologous human acrocentric chromosomes.

5.3 Compute environment

We applied the nf-core/pangenome pipeline v1.1.2 to various inputs evaluating both the scalability of the all-vs-all alignment step as well as the pipeline as a whole. We used Nextflow version 23.10.1.5891 and Singularity version 3.8.7-1.el8 for each pipeline run. Experiments were conducted on our core facility cluster (CFC) with 24 Regular nodes (32 cores / 64 threads with two AMD EPYC 7343 processors with 512 GB RAM and 2 TB scratch space) and 4 HighMem nodes (64 cores / 128 threads with two AMD EPYC 7513 processors with 2048 GB RAM and 4TB scratch space). Each Nextflow process was given at most 64 threads. This ensures a fair run time comparison with PGGB v0.5.4 which was always executed on one Regular node via Slurm.

5.4 Estimation of the carbon footprint of pipeline runs

We also estimated the carbon dioxide equivalent (CO_2e) emissions of each nf-core/pangenome pipeline run using the nf-co2footprint Nextflow plugin (https://github.com/nextflow-io/nf-co2footprint, last accessed October 2024) v1.0.0-beta. Using the Nextflow resource usage metrics and information about the power consumption of the compute system, the plugin first estimates the energy consumption for each pipeline task. It then uses the consumed energy's location-specific carbon intensity to estimate the respective CO_2e emission. The calculations are based on the carbon footprint computation method developed in the Green Algorithms project (www.green-algorithms.org) (Lannelongue *et al.*, 2021).

5.5 Alignment jobs distribution

The computationally heavy all versus all base-pair level alignments can be distributed across nodes of a cluster: First, WFMASH is run in mapping mode (WFMASH MAP), finding all sequence homologies using approximate alignments. The resulting Pairwise mApping Format (PAF) file is split into chunks of equal problem size. The number of chunks is manually selected. The value can be guided by the number and size of the input sequences, and by the available hardware. Assuming the number of chunks equals the number of nodes on a cluster, then potentially each base-pair level alignment (WFMASH ALIGN) can be run in parallel on each node (Fig. 1a, cyan tubes). All resulting PAFs are then forwarded to the pipeline's core workflow which is continued at the SEQWISH process.

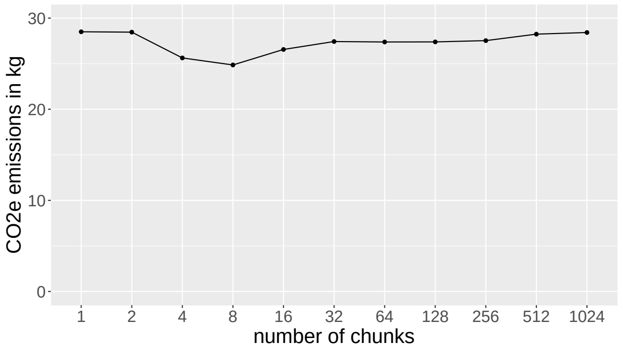

Fig. S1. Base-pair level alignment evaluation. CO_2e emissions are stable across varying numbers of chunks.

5.6 1KGP chromosome 19 data set

The FASTA of the chromosome 19 data set was downloaded in December 2023 from http://dolomit.cs.tu-dortmund.de/chr19.1000.fa.xz. The data

Sample Name	Length	Nodes	Edges	Paths	Components	A	C	T	G	N
**chr19.1000**	3 393 915 450	2 594 408	3 498 791	1 000	1	19 633 450	16 968 263	19 854 852	17 458 885	3 320 000 000
**chr19.1000.crush**	73 921 450	2 594 408	3 498 791	1 000	1	19 633 450	16 968 263	19 854 852	17 458 885	6 000

Fig. S2. Screenshot of the output of ODGI’s MultiQC module displaying the vital graph statistics calculated by odgi stats of the 1000 Genomes Project 1000 haplotypes chromosome 19 pangenome graphs. In the crushed graph consecutive Ns of all nodes containing Ns were merged into just one N per node. A: Number of adenine bases in the graph. C: Number of cytosine bases in the graph. T: Number of thymine bases in the graph. G: Number of guanine bases in the graph. N: Number of bases with unknown base identity.

Fig. S3. odgi draw 2D layout displaying the graph topology of the crushed 1KGP pangenome graph. Structural variation would appear as bubbles.

set is described in (Kuhnle *et al.*, 2020). Statistics of the built pangenome graph can be seen in Supplementary Fig. 5.6. The initial graph contains over 97% of Ns. We applied *odgi crush* (odgi version 0.8.6), which crushes consecutive Ns of all nodes containing Ns into just one N per node, to the 1KGP chromosome 19 pangenome graph. This brings down the number of Ns from over 3B to exactly 6000 (Suppl. Fig. 5.6). The 2D visualization (Suppl. Fig. 5.6) is perfectly linear without any large SVs present hinting that only short-read data was used to create the haplotype sequences. In contrast, the 2D layout of the HPRC PGGB chromosome 19 pangenome graph (Heumos *et al.*, 2024) clearly presents SVs, especially in the centromere’s location. Investigating these complex regions of a human chromosome is only possible when using long-read assemblies for graph construction.

5.7 *E.coli* data set

The 2146 full length E. coli sequences originate from Genbank (Sayers *et al.*, 2021) and were downloaded 18 months ago. The initial pangenome consisted of 2 graphical components (Suppl. Fig. 5.7). This means that no strong homologies were found in some sequences. There can be many reasons for additional graph components: (a) The chosen sequence identity during the WFMASH mapping was not low enough. Although we went for a low 90% sequence identity (as was done by Garrison *et al.* (2023)), we still observe this additional graph component, so its sequence must be quite dissimilar to all other sequences. (b) There is human contamination in the bacterial sequences (Breitwieser *et al.*, 2019). (c) Some sequences from GenBank may be of a low quality or were misassembled. We then used odgi explode to extract the largest graphical component, applied *odgi crush* and dropped all paths containing “plasmid” in their path name with *odgi paths*. This left us with one component and 2013 paths. In the 2D visualization, we observe a highly connected graph (Suppl. Fig. 5.7). All the reasons mentioned above, but especially horizontal gene transfer could explain this phenomenon. Therefore, there are a lot of edge crossings in the pangenome graph. The long stretches is dangling sequence. We speculate that here the 88 thousand Ns could play role.

Sample Name	Length	Nodes	Edges	Paths	Components	A	C	T	G	N
ecoli_2146	140 465 404	6 118 071	8 972 252	2 146	2	21 671 24[illegible]	19 111 201	21 699 33	19 131 791	58 851 837
ecoli2146.pan.explode	140 321 750	6 113 871	8 966 644	2 143	1	21 631 63[illegible]	19 077 54[illegible]	21 663 73	19 096 998	58 851 837
ecoli2146.pan.explode.crush	81 557 898	6 113 871	8 966 644	2 143	1	21 631 63[illegible]	19 077 54[illegible]	21 663 73	19 096 998	87 985
ecoli2146.pan.explode.crush.no_plasmids	81 557 898	6 113 871	8 966 644	2 013	1	21 631 63[illegible]	19 077 54[illegible]	21 663 73	19 096 998	87 985

Fig. S4. Excerpt of the 2146 sequences E. coli pangenome graph's MultiQC report. Displayed are vital graph statistics by MultiQC's ODGI module. A: Number of adenine bases in the graph. C: Number of cytosine bases in the graph. T: Number of thymine bases in the graph. G: Number of guanine bases in the graph. N: Number of bases with unknown base identity.

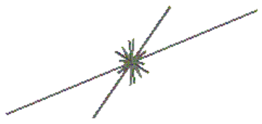

Fig. S5. odgi draw 2D layout visualization of the 2013 haplotypes E. coli pangenome graph.
